# Endovascular Excimer Laser-Assisted Balloon Angioplasty for Infrapopliteal Arteries in Thromboangiitis Obliterans: A Treatment for Acute-Phase TAO

**DOI:** 10.3389/fcvm.2022.831340

**Published:** 2022-03-04

**Authors:** Hongji Pu, Yihong Jiang, Zhaoyu Wu, Jiahao Lei, Jiateng Hu, Peng Qiu, Xing Zhang, Qun Huang, Xinwu Lu, Minyi Yin, Zhen Zhao

**Affiliations:** Department of Vascular Surgery, Shanghai Ninth People's Hospital, Shanghai JiaoTong University School of Medicine, Shanghai, China

**Keywords:** thromboangiitis obliterans (TAO), Buerger's disease, excimer laser, angioplasty, endovascular treatment

## Abstract

**Background:**

Thromboangiitis obliterans (TAO, Buerger's disease) is an inflammatory and obstructive vasculopathy, which leads to limb ischemic rest pain and ulcerations in the acute stage.

**Objectives:**

This study aimed to assess the feasibility of excimer laser-assisted balloon angioplasty (BA) for patients with acute infrapopliteal TAO.

**Method:**

This was a single-center retrospective cohort study. In this study, 220 patients with 210 target limbs between January 2012 and September 2021 were involved. Among them, 52 target limbs have received endovascular excimer laser-assisted balloon angioplasty from January 2017. The ankle brachial index (ABI), rest pain score, ulcer, Rutherford classification, and TASC II classification were assessed. The follow-up time was 6 months.

**Results:**

The technical success rate of laser + BA and BA groups was 71.15 and 65.82% (*p* = 0.5021), respectively. After intervention, the ABI of two groups were 0.85 ± 0.20 and 0.77 ± 0.20 (*p* = 0.0419), and the rest pain score was 1.00 ± 1.43 and 1.71 ± 2.25 (*p* = 0.0449). During the 6 months follow-up, the ABI of two groups was 0.76 ± 0.17 and 0.75 ± 0.23 (*p* = 0.8539), the rest pain score was 1.43 ± 1.82 and 2.24 ± 2.06 (*p* = 0.0783), and the ulcer rate was 23.68 and 40.98% (*p* = 0.0867), respectively. The proportion of patients who were assessed as TASC II C/D or Rutherford 4–6 in laser +BA group was significantly lower than that in BA group, indicating that the former had better efficacy. The rate of critical limb ischemia and restenosis in the laser +BA group was lower than that in the BA group (47.36 vs. 67.22%; 21.05 vs. 34.43%) during follow-up. In the laser + BA group, the reintervention rate was lower than that in the BA group (2.70 vs. 8.20%, *p* = 0.0425). No serious adverse events (AEs) occurred.

**Conclusion:**

Excimer laser debulking-assisted angioplasty is a feasible, effective, and safe method to treat acute infrapopliteal TAO.

## Introduction

Thromboangiitis obliterans (TAO, Buerger's disease) is a non-atherosclerotic segmental inflammatory vascular disease commonly affecting the small- and medium-sized vessels of extremities (1). The basic pathology of TAO is that inflammatory thrombus of the vascular wall leads to arterial stenosis and occlusion (1, 2). Male sex, smoking, and limb ischemia before the age of 45 years are the known risk factors (2). As stenosis and occlusion progress, patients suffer from intermittent claudication, resting pain, ulcers, tissue loss, and eventually amputation. The limited treatment of TAO includes strict smoking cessation, medication, and surgical operation often with no acceptable target vessel for bypass. Although endovascular intervention, such as balloon angioplasty (BA) with local vasodilators improved lower limb ischemia, the treatment is still with great challenges, such as high complication rate, inability to pass through the lesion, and low patency rate when infrapopliteal arteries are involved (3, 4).

The pathological features of TAO contribute to a significant decline in the patency rate after the endovascular intervention. The course of TAO was divided into the acute phase, subacute (intermediate) phase, and chronic (end-stage) phase. In the acute phase, patients present typical Buerger resting pain and ulcerations on the toes or feet. The acute phase is characterized by occlusive, highly cellular, and inflammatory thrombi in the lumen mixed with fibrosis (1, 5). Another feature of TAO is that it mostly affects blood vessels in the distal extremities. Currently, it remains difficult to treat small- and medium-sized arterial occlusions, and it is considered one of the most refractory types of limb ischemia in the field of surgery. A previous meta-analysis of infrapopliteal angioplasty suggested that the patency of vessels after endovascular treatment was 77.4 ± 4.1% at 1 month and only 48.6 ± 8.0% at 36 months (6). These characteristics limited suitable treatment for lower limb TAO in the past (7–9).

In recent years, as the atherectomy was used in the coronary and peripheral arteries, the patency was improved obviously (10). However, the feasibility and efficiency of excimer laser atherectomy for patients with acute infrapopliteal TAO remains unknown. Excimer laser therapy may be a solution for infrapopliteal arterial occlusions dominantly caused by intraluminal obstruction, which has been used in patients with critical limb ischemia who are unfit for traditional revascularization for 20 years (11, 12). While laser therapy is considered to be suitable for endovascular volume reduction in infrapopliteal arteries, there are few studies on whether it is effective for acute-phase TAO. To address this issue, we conducted this retrospective cohort study.

The objective of this study was to evaluate the effectiveness and safety of excimer laser debulking combined with BA in patients with acute-phase TAO. All patients who underwent laser + BA or BA alone combined with local drug perfusion had limb ischemic symptoms and tibioperoneal arterial occlusion. CT angiography (CTA), digital subtraction angiography (DSA), or intravenous ultrasound (IVUS) was used to measure the lesion and the efficacy of laser-assisted angioplasty.

## Methods

### Study Design and Patient Population

This was a retrospective study of severe TAO patients with tibioperoneal arterial occlusion. Between January 2012 and September 2021, 210 consecutive patients who met the criteria were involved in the study. Among them, excimer laser-assisted BA was conducted in 52 target limbs from January 2017. The diagnostic and exclusive criteria of TAO are listed below. The study was approved by the Ethics Committee of Shanghai JiaoTong University, School of Medicine (Shanghai, China), and informed consent was obtained.

The diagnostic criteria included: (1) exclusion of other vasculitis or hypercoagulable states by investigations for rheumatoid factor and lupus anticoagulants or serologic analyses; (2) onset before the age of 50 years; (3) partial infrapopliteal arterial occlusions; (4) iliac and/or superficial femoral artery involvement, and either upper limb involvement or phlebitis migrants; and (5) the absence of atherosclerotic risk factors (such as, hypertension, diabetes mellitus, hyperlipidemia, or cardiovascular disease) other than smoking; and (6) patients voluntarily chose excimer laser debulking and agreed with the publication of relevant clinical data. The exclusion criteria were as follows: (1) patients under 18 years old who declined to participate or were not in the acute phase on admission; (2) patients with other extremity arterial diseases, such as atherosclerosis obliterans and Takayasu's arteritis; (3) patients in whom at least one of the anterior tibial arteries, posterior tibial arteries, and peroneal arteries were not affected; and (4) patients with the presence of extremity ulcers or tissue loss due to non-arterial causes, such as fungal infection and tubercular ulcers.

### Procedures

All patients were prepared according to the clinical guidelines, with the administration of anticoagulants and vasodilators. During the perioperative period, anticoagulants and vasodilators were routinely used, such as the injections of low molecular weight heparin, papaverine, and oral sarpogrelate. After surgery, patients were treated with oral rivaroxaban 10 mg per day for 3 months since 2017 and with clopidogrel 75 mg per day and sarpogrelate 100 mg ter in die for at least 6 months. CTA was used for the diagnosis and outpatient follow-up. DSA was performed as diagnostic and guidance imaging pre- and peri-procedurally. *Via* DSA, the artery with the distal outflow tract of the anterior tibial artery, posterior tibial artery, or peroneal artery was considered the target artery. IVUS was used to evaluate the postoperative acquisition of lumen diameter and vessel wall thickness of the operated arteries in some cases.

The guidewire passes through the narrow or occluded segment to the distal outflow tract with standard endovascular techniques. For the target artery, an excimer laser system (CVX-300 excimer laser system, Philips, Netherlands) was used from the bifurcation of the popliteal artery to the ankle. A 0.9–2.0 mm laser catheter was conducted over the guidewire and advanced at a speed of less than 0.5–1 mm/s 2–3 times with the energy (30–60 mJ/mm^2^)/(25–80 Hz), according to the instrument and the lesion characteristics. After the lumen was obtained, BA with a 1.5–3.0 mm diameter balloons was performed in the target artery. For lesions resistant to laser catheters, 1.5 mm diameter balloon angioplasty was attempted to temporarily dilate the artery before atherectomy. In two groups, vasodilators, such as 0.5 mg nitroglycerin or 30 mg papaverine, and 100,000–200,000 units of urokinase were used through the catheter above the target artery. When both the laser catheter and the balloon failed to pass through the artery, the combined intervention was considered a failure, then, vasodilators and urokinase were administrated.

### Follow-Up and Outcomes

The outcomes included ankle brachial index (ABI), rest pain score, ulcer healing, Rutherford classification, TASC II classification, reintervention, tissue loss, and total amputation. All indices were measured before and 6 months after endovascular treatment. The ABI was calculated by the systolic pressure at the ankle divided by the systolic pressure at the arm. Rest pain was assessed by patients rating their pain on a scale of 0–10. Adverse events (AEs) were continuously monitored during follow-up. The follow-up time was 6 months.

### Statistical Analysis

Continuous data are expressed as the mean ± SD and were compared using Student's *t*-test. Binary data are expressed as absolute or relative frequencies and were compared by the chi-square or Fisher's exact tests. Ranked data are expressed as absolute values and were compared by the Cochran-Armitage trend test. The patency rate was calculated by Kaplan–Meier estimator. For all tests, *p* < 0.05 was defined as significant. All analyses were performed using SAS 9.2 (Stat, SAS Institute, Cary, NC, USA).

## Results

### Characteristics of Patients

A total of 210 patients with 210 target limbs were involved. Fifty-two of them received laser + BA, and 158 of them received BA alone. In the laser + BA group, 37 patients successfully received laser-assisted angioplasty (technical success rate 71.15%), while the technical success rate of the BA group was 65.82%. The general patient characteristics are listed in Table 1. There was no significant difference in demographics or disease severity between the laser + BA and BA groups, and no significant difference was observed between all involved patients and patients who received successful angioplasty.

**Table 1:** The general characteristics of involved patients.

**Variable**	**Laser + BA**			**BA**			***p*** **value**
	**Total** **(***n*** = 52)**	**Technical success** **(***n*** = 37)**	***p*** **value**	**Total** **(***n*** = 158)**	**Technical success** **(***n*** = 104)**	***p*** **value**	
Male	75.00% (39)	73.68% (28)	0.6500	77.22% (122)	78.85% (82)	0.1474	0.1592
Age	34.67 ± 6.83	34.00 ± 7.32	0.6577	36.88 ± 9.14	36.75 ± 9.27	0.9110	0.1112
Diabetes	0 (0)	0 (0)	1.0000	0 (0)	0 (0)	1.0000	1.0000
Hypertension	0 (0)	0 (0)	1.0000	0 (0)	0 (0)	1.0000	1.0000
Dyslipidemia	0 (0)	0 (0)	1.0000	0 (0)	0 (0)	1.0000	1.0000
CAD	7.69% (4)	8.11% (3)	0.9717	4.43% (7)	1.92% (2)	0.3254	0.1191
CKD	0 (0)	0 (0)	1.0000	1.24% (2)	1.87% (2)	>0.9999	>0.9999
Smoking							
Never	34.62% (18)	43.24% (16)	0.4612	44.94% (71)	46.15% (48)	0.9753	0.0671
Former	26.92% (14)	16.22% (6)		32.91% (52)	32.69% (34)		
Current	38.46% (20)	40.54% (15)		22.15% (35)	21.15% (22)		
ABI	0.70 ± 0.20	0.75 ± 0.20	0.2600	0.70 ± 0.21	0.72 ± 0.22	0.4245	0.9959
Rest pain score	4.87 ± 1.22	4.89 ± 1.31	0.9222	4.49 ± 1.50	4.57 ± 1.57	0.6794	0.1022
Ulcer	51.92% (27)	56.76% (21)	0.8319	50.96% (53)	46.30% (25)	0.8996	0.8731
Rutherford							
0	0 (0)	0 (0)	0.9357	0 (0)	0 (0)	0.9239	0.9321
1	0 (0)	0 (0)		0 (0)	0 (0)		
2	0 (0)	0 (0)		0 (0)	0 (0)		
3	0 (0)	0 (0)		0 (0)	0 (0)		
4	48.08% (25)	45.95% (17)		50.63% (80)	49.04% (51)		
5	28.85% (15)	32.43% (12)		28.48% (45)	30.77% (32)		
6	23.08% (12)	21.62% (8)		20.89% (33)	20.19% (21)		
TASC II							
0	0 (0)	0 (0)	0.7225	0 (0)	0 (0)	>0.9999	>0.9999
A	0 (0)	0 (0)		0 (0)	0 (0)		
B	0 (0)	0 (0)		0 (0)	0 (0)		
C	46.15% (24)	48.65% (18)		46.84% (74)	46.15% (48)		
D	53.85% (28)	51.35% (19)		53.16% (84)	53.85% (56)		
Previous angioplasty	13.46% (7)	13.51% (5)	0.9666	10.76% (17)	10.58% (11)	>0.9999	0.6181
Previous bypass	3.85% (2)	5.41% (2)	0.7473	3.80% (6)	3.85% (4)	>0.9999	>0.9999
Previous amputation	11.54% (6)	10.81% (4)	0.9049	11.39% (18)	4.81% (5)	0.0761	>0.9999

*The data were expressed as mean ± SD or percentage (number). CAD, coronary artery disease; CKD, chronic kidney disease*.

### Characteristics of the Laser-Assisted Angioplasty Procedure

**Figure 1:**
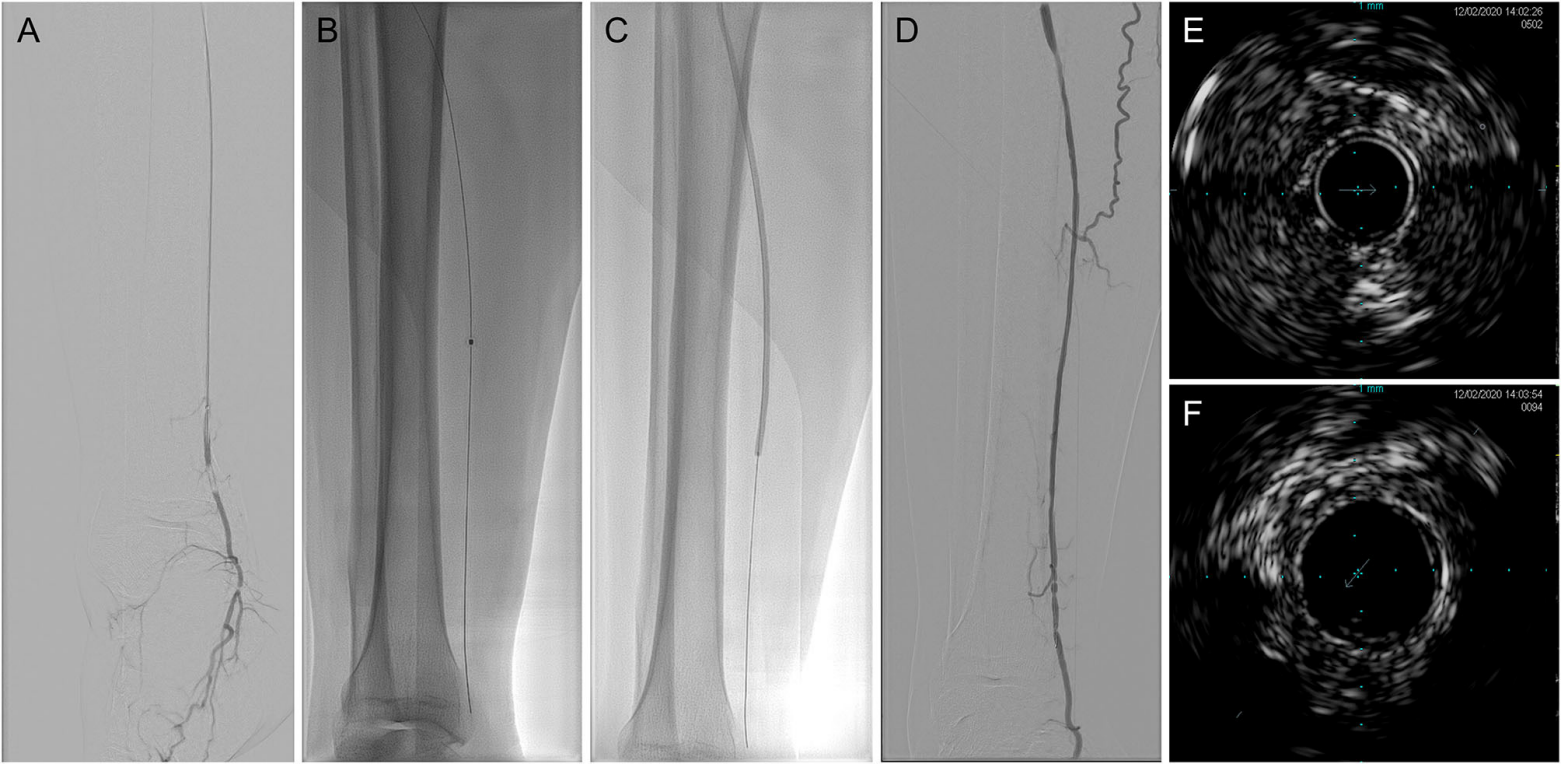
Representative digital subtraction angiography (DSA) and intravenous ultrasound (IVUS) imaging of laser-assisted angioplasty intervention. **(A)** Baseline angiography; **(B)** excimer laser angioplasty; **(C)** balloon angioplasty (BA); **(D)** after intervention angiography; **(E)** baseline IVUS imaging; and **(F)** after intervention IVUS imaging. DSA, digital subtraction angiography; IVUS, intravenous ultrasound.

The procedural data of laser-assisted BA are summarized in Table 2. There was no significant difference in lesion length or target artery distribution between the technical success and technical failure groups. The average lesion length in patients who received the successful intervention was 34.05 ± 9.63 (range: 15–50) cm. Among the target arteries, the proportions of the anterior tibial artery, posterior tibial artery, and peroneal artery in the technical success group were 45.95, 43.24, and 10.81%, respectively. In contrast, the proportion of peroneal arteries in the technical failure group was larger than that in the technical success group (without significance, *p* = 0.0525). The mean maximum fluency was 52.84 ± 4.00 (range: 45–55) mJ/mm^2^, and the total range was 30–60 mJ/mm^2^. The mean maximum repetition rate was 72.57 ± 6.83 (range: 60–80) Hz, and the total range was 25–80 Hz. After the intervention, the minimum vessel wall thickness measured by IVUS was 1.10 ± 0.29 (range: 0.52–1.53) mm. No artery perforation or distal embolization was observed. The representative case is shown in Figure 1.

**Table 2:** The procedural data of laser-assisted angioplasty.

**Variable**	**Technical success** **(***n*** = 37)**	**Technical failure** **(***n*** = 15)**	***p*** **value**
Lesion length (cm)	34.05 ± 9.63 (15–50)	34.33 ± 7.98 (18–48)	0.9214
Target arteries			
Anterior tibial artery	45.95% (17)	33.33% (5)	0.0525
Posterior tibial artery	43.24% (16)	26.67% (4)	
Peroneal artery	10.81% (4)	40.00% (6)	
Serious adverse events			
Artery perforations	0 (0)		
Distal embolization	0 (0)		
Laser therapy			
The maximum fluency (mJ/mm^2^)	52.84 ± 4.00 (45–55)		
The maximum repetition rate (Hz)	72.57 ± 6.83 (60–80)		
The minimum vessel wall thickness after intervention (mm)	1.10 ± 0.29 (0.52–1.53)		

*The data were expressed as mean ± SD or percentage (number)*.

### Outcomes

The outcomes of the laser + BA group and BA group are summarized in Table 3. Both laser + BA group and BA treatment improved the ABI, rest pain score, and arterial patency of patients. The ABI of the laser + BA group was higher (0.84 ± 0.19 vs. 0.77 ± 0.17, *p* = 0.0463), and the rest pain score were lower (1.00 ± 1.43 vs. 1.71 ± 2.25, *p* = 0.0449) than these of BA group with significance. The TASC II classification in laser + BA group is lower (0: 100%) compared with that in BA group (0: 76.92%, A: 8.65%, B: 6.73%, C: 7.69%, and D: 0).There was a significant difference in the distribution of TASC II (*p* = 0.0006) classification between the two groups. No residual stenosis was reported in the laser + BA group while only 76.92% of patients in the BA group reported no residual stenosis.

**Table 3:** The clinical outcomes of patients with TAO received laser-assisted angioplasty and BA alone.

**Variable**	**Immediately after intervention**			**6 months after intervention**		
	**Laser + BA**	**BA**	***p*** **value**	**Laser + BA**	**BA**	***p*** **value**
	**(*n =* 37)**	**(*n =* 104)**		**(*n =* 37)**	**(*n =* 61)**	
ABI	0.85 ± 0.20	0.77 ± 0.20	0.0419	0.76 ± 0.17	0.75 ± 0.23	0.8539
Rest pain score	1.00 ± 1.43	1.71 ± 2.25	0.0449	1.43 ± 1.82	2.24 ± 2.06	0.0783
Ulcer				23.68% (9)	40.98% (25)	0.0867
Rutherford classification					
0				39.47% (15)	26.23% (16)	0.0306
1				7.89% (3)	1.64% (1)	
2				0 (0)	1.64% (1)	
3				2.63% (1)	3.28% (2)
4				23.68% (9)	26.23% (16)	
5				23.68% (9)	29.51% (18)	
6				0 (0)	11.48% (7)	
TASC II classification					
0	100% (37)	76.92% (80)	0.0006	78.95% (30)	65.57% (40)	0.0224
A	0 (0)	8.65% (9)		2.63% (1)	6.56% (4)	
B	0 (0)	6.73% (7)		15.79% (6)	3.28% (2)	
C	0 (0)	7.69% (8)		0 (0)	16.39% (10)	
D	0 (0)	0 (0)		0 (0)	8.20% (5)	
Non-SAE	0 (0)	0 (0)	1.0000	0 (0)	0 (0)	1.0000
SAE	0 (0)	0 (0)	1.0000	0 (0)	0 (0)	1.0000
Reintervention				2.70% (1)	8.20% (5)	0.0425
Tissue loss				0 (0)	8.20% (5)	0.1533
Amputation				0 (0)	3.28% (2)	0.5251

*BA, balloon dilation; SAE, serious AE, defined as artery perforation, iatrogenic embolization, other hospitalization, and death; TAO, thromboangiitis obliterans*.

All patients (100%) who received successful laser-assisted angioplasty and 61 patients (58.7%) who received successful BA completed follow-up visits (range: 3–9 months). The ABI of the laser + BA group was higher than that of the BA group (0.76 ± 0.17 vs. 0.75 ± 0.23, *p* = 0.8539) without significance. Compared with patients in the BA group, patients in the laser + BA group reported lower rest pain scores (1.43 ± 1.82 vs. 2.24 ± 2.06, *p* = 0.0783) and ulcer rates (23.68 vs. 40.98%, *p* = 0.0867), with no significance. The TASC II classification in laser + BA group is lower (0: 78.95%, A: 2.63%, B: 15.79%, C: 0, and D: 0) compared with that in BA group (0: 65.57%, A: 6.56%, B: 3.28%, C: 16.39%, and D: 8.20%). The mean Rutherford classification in laser + BA group is lower (0: 39.47%, 1: 7.89%, 2: 0, 3: 2.63%, 4: 23.68%, 5: 23.68%, and 6:0) compared with that in BA group (0: 26.23%, 1: 1.64%, 2: 1.64%, 3: 3.28%, 4: 26.23%, 5: 29.51%, and 6: 11.48%). There were significant differences in the distribution of Rutherford (*p* = 0.0306) and TASC II (*p* = 0.0224) classification between the two groups.

**Figure 2:**
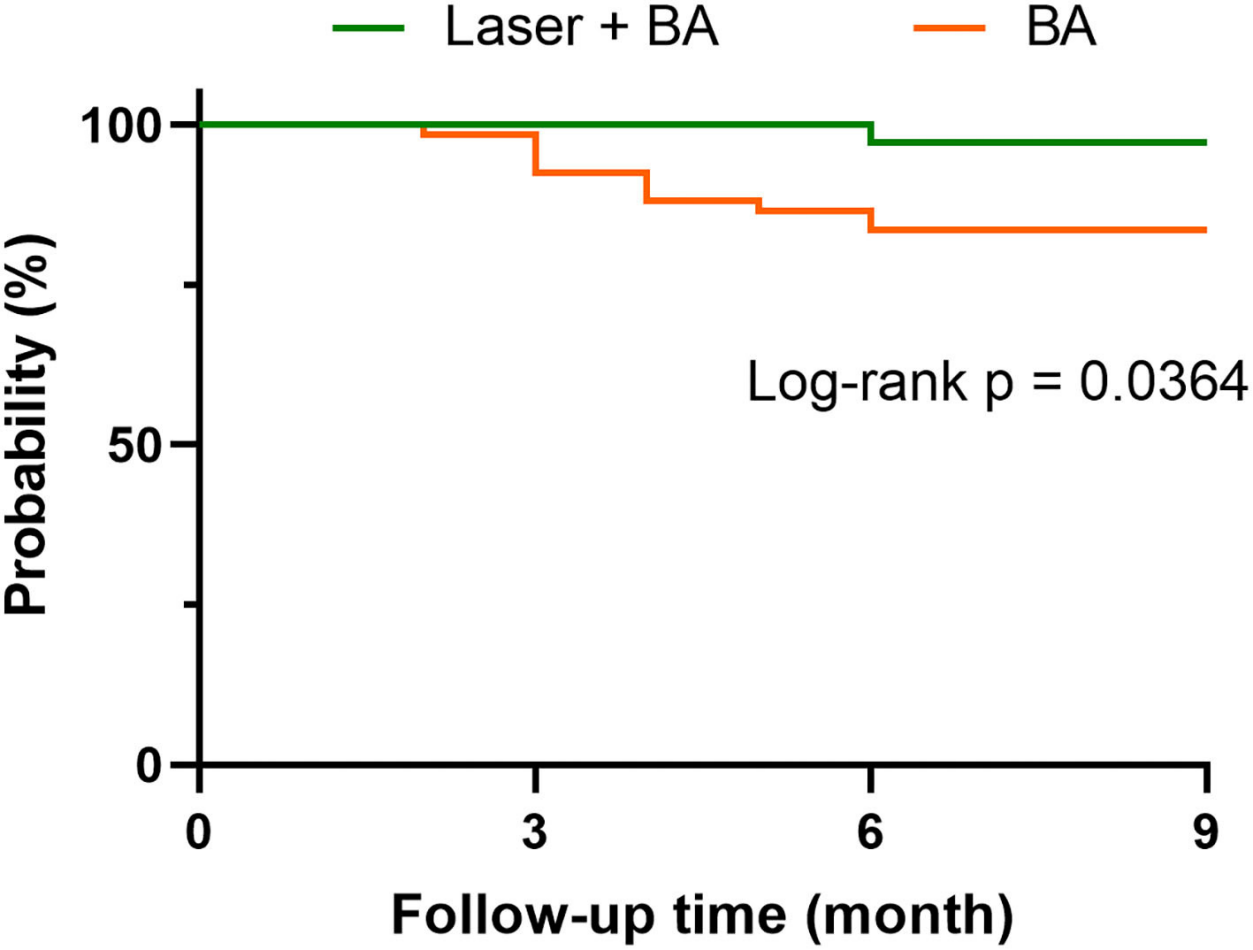
The free from rehospitalization curves of laser-assisted angioplasty and BA groups. The case of rehospitalization includes reintervention, tissue loss, and amputation.

All patients who received the successful laser + BA intervention had no residual stenosis in the treated artery immediately after the intervention. According to CTA results at follow-up, TASC II A/B stenosis reappeared in 7 patients during the follow-up (no TASC II C/D occurred). Among the 7 patients, 1 patient received reintervention, and the other 6 patients had no reintervention plan due to pain relief and ulcer healing. No tissue loss or amputation was reported. In the BA group, 21 patients reported stenosis according to CTA (15 of them were TASC II C/D), and 5 patients received reintervention. In addition, 5 patients reported tissue loss, and 2 patients underwent amputation. According to the Kaplan–Meier curves, the free from rehospitalization rate of laser + BA group was significantly higher than that of BA alone group (*p* = 0.0364). The cases of rehospitalization included reintervention, tissue loss, and amputation (Figure 2).

Concerning the problem that the follow-up rate in the BA group was not very substantial, we compared the demographics of the follow-up population and those lost to follow-up. There was no difference in sex, age, history of disease and smoking, or severity of TAO. Based on this, the main conclusions were unaffected.

Regarding AEs of laser-assisted angioplasty, 4 minor complications after laser-assisted angioplasty were reported (3 cases of pain at the puncture site and 1 case of calf swelling), and all complications were resolved prior to discharge. No complications were reported during follow-up. No serious AEs (defined as artery perforation, iatrogenic embolization, other hospitalizations, or death) were reported. In the BA group, 13 minor complications after intervention were reported (6 cases of calf swelling, 5 cases of pain at the puncture site, 2 cases of pseudoaneurysm at the puncture site, and 2 cases of calf discomfort). No complications were reported during follow-up and no serious AEs were reported.

## Discussion

In this study, 210 TAO patients with infrapopliteal arterial occlusion were enrolled. Fifty-two of them planned to receive 308 nm excimer laser-assisted BA in the target tibioperoneal artery, while 37 patients successfully received laser-assisted angioplasty (technical success rate 71.15%). The patency rate was 100% immediately after the intervention, and through the 6-month follow-up, the patency rate was 81.08%. No serious AE occurred during the follow-up. Compared with those of the BA group, the perfusion index (ABI and TASC II classification) of the laser + BA group was significantly better. More importantly, the Rutherford classification and reintervention rate of the laser + BA group during the follow-up was significantly lower than that of the BA group.

According to current clinical studies and practice, there is no ideal revascularization approach for patients with TAO. In the past 70 years, neither autologous vein grafts nor sympathectomy had satisfactory results. In recent years, the focus has turned to endovascular therapy. Balloon dilation, drug-coated balloon, local injection of vasodilators, and stent implantation are gradually applied to TAO. However, some retrospective studies have reported that BA is a feasible method with a relatively high technical success rate, but the patency rate and reintervention-free rate need to be improved. According to previous clinical studies, the patency rate of BA during the 6–48-month follow-up was 14.37–60.2%, and the reintervention rate was 28.3–62.8% (13–16). Furthermore, it is difficult to conduct endovascular debulking in the infrapopliteal arteries as smaller lumen diameters compared with femoral-popliteal arteries.

The excimer laser, with a wavelength of 308 nm, is considered to be safe for ablating atherosclerotic plaques and thrombi with fibrosis in the arterial lumen (10–12). For infrapopliteal arterial occlusions, laser therapy has advantages. A previous randomized controlled trial involving 250 patients reported that patients with infrapopliteal arterial occlusion benefited from excimer laser combined debulking with balloon dilation. Compared with BA alone, laser + BA has higher free from revascularization rate (73.5 vs. 51.8%, *p* < 0.005) and lower AE rate (5.8 vs. 20.5%, *p* < 0.001) (17). In this study, an excimer laser was used to ablate occlusion in arteries affected by TAO. Two of the major pathological changes in TAO were acute thrombotic obstruction of medium- and small-sized arteries and chronic organization of the thrombus with the intimal hyperplasia. The former might lead to blood supply loss in the extremities, and the latter is the cause of pathological remodeling of the affected arteries (1, 4). Since an excimer laser can be used as a debulking system in infrapopliteal arteries, laser therapy might be an option for rapid vascularization in the acute phase and may prevent luminal restenosis in the long term.

After 0.018 or 0.014 guidewire access the calf artery occlusion, 0.9–2.0 mm laser catheters were used and advanced at a speed of less than 0.5–1 mm/s. The mean maximum fluency was 52.84 ± 4.00 mJ/mm^2^, and the mean maximum repetition rate was 72.57 ± 6.83 Hz. Before laser debulking, pre-dilation with a small diameter balloon facilitates the passage of the laser catheter through the lesion occasionally. The balloon pre-dilation also enables the laser catheter to pass through the arterial lumen at a uniform speed, which may be beneficial to reduce the occurrence of complications, such as perforation and dissection. In addition, the high-speed advancement of the laser catheter might lead to incomplete debulking. It is not recommended to advance the laser catheter at a speed faster than 1 mm/s. Our preliminary results showed that the laser debulking is effective for acute-phase TAO. Although BA could improve the perfusion index, the ischemic symptoms and arterial patency of patients, laser + BA as an innovative treatment showed more efficacy particularly in ABI improvement, rest pain relief, and arterial patency in this study. However, there was no statistical significance in ulcer healing rate and rest pain score at the 6-month follow-up between the two groups. Compared with open surgery, endovascular therapy is less traumatic and can be applied to patients who are unsuitable for bypass (15), which also has better efficacy than the only application of vasodilators. Compared with balloon dilation or stent implantation, laser + BA may be a therapy with an ideal technical success rate and curative effect once the guidewire passes through the occlusion.

Limitations of this study remain to be addressed. The major shortcoming is that the patient population is relatively small. One of the critical reasons is that CVX-300 excimer laser catheter is expensive for most patients with TAO and does not enter the scope of medical insurance. Another reason is that TAO in the acute phase is difficult to diagnose. Although smoking history, phlebitis history, and onset before 50 years old are considered diagnostic criteria, there are no specific laboratory or imaging tests to aid in the diagnosis of thromboangiitis obliterans (1, 4). The current diagnosis of TAO is a diagnosis of exclusion, which makes the rate of missed diagnosis high and makes it difficult for this study to include patients with TAO. To solve this issue, the extension of the study duration was required to include more study samples. In addition, the follow-up rate of the control group was not substantial. The main reason is that this is a retrospective study, and the patients who underwent BA treatment were not under active follow-up. As this study continues, patients who underwent BA only will also be enrolled in the follow-up cohort.

Another prospect of laser therapy is for infrapopliteal arterial occlusion. At present, a 308 nm excimer laser is utilized in the revascularization of complex atherosclerotic obliterans (ASO) (18) and diabetic vascular diseases (10). For lesions resistant to guidewires, the laser was able to ablate plaques and thrombi, and thus, the apparatus was able to ablate the lesions through occlusion (the “step-by-step” technique) (18, 19). In the acute phase of TAO, there are also cases where the thrombus mixed with chronic lesions completely obstructs the tract and is resistant to the balloon. Based on the technique employed for ASO, laser therapy might be applied to arteries in patients with TAO based on furthermore evidence.

## Conclusion

Excimer laser debulking-assisted BA in acute-phase TAO is an innovative method. Our data provide promising findings that may offer a chance for acute TAO treatment to increase patency rate, resting pain relief, and accelerate ulcer healing, compared with BA alone. Further study is warranted to obtain follow-up data on the long-term effectiveness of excimer laser treatment in acute-phase TAO.

## Impact on Daily Practice

Excimer laser-assisted balloon angioplasty (BA) is a feasible and safe treatment for patients with “no-option” infrapopliteal arterial thromboangiitis obliterans (TAO). This combined intervention achieved technical success in the majority of patients, and the results indicated that it was safe and contributed to patency rate increases, rest pain relief, and ulcer healing.

## Data Availability Statement

The raw data supporting the conclusions of this article will be made available by the authors, without undue reservation.

## Ethics Statement

The studies involving human participants were reviewed and approved by the Ethics Committee of Shanghai JiaoTong University, School of Medicine (Shanghai, China). The patients/participants provided their written informed consent to participate in this study. Written informed consent was obtained from the individual(s) for the publication of any potentially identifiable images or data included in this article.

## Author Contributions

ZZ, MY, and XL contributed to the study concept, research design, perform the research, and supervised the study. HP, YJ, and ZW participated in data collections, statistical analysis, and interpretation of data. PQ, XZ, and QH participated in data collections. HP and ZZ drafted the manuscript. MY, JL, and JH revised the manuscript. XL is for the interpretation of this study. All authors read and approved the final manuscript.

## Funding

This work was supported by grants from the National Natural Science Foundation of China (82070449, 81971758, and 82170509).

## Conflict of Interest

The authors declare that the research was conducted in the absence of any commercial or financial relationships that could be construed as a potential conflict of interest. The handling editor YZ declared a shared affiliation with several of the authors HP, YJ, ZW, JL, JH, PQ, XZ, QH, XL, MY and ZZ at time of review.

## Publisher's Note

All claims expressed in this article are solely those of the authors and do not necessarily represent those of their affiliated organizations, or those of the publisher, the editors and the reviewers. Any product that may be evaluated in this article, or claim that may be made by its manufacturer, is not guaranteed or endorsed by the publisher.
